# Cell Phone Ownership and Cellular Text/Email Capabilities Among Temporary and Payroll Construction Workers

**DOI:** 10.3389/fpubh.2020.00042

**Published:** 2020-03-12

**Authors:** Alberto J. Caban-Martinez, Kevin J. Moore, Juanita J. Chalmers, Katerina M. Santiago, Melissa Baniak, Melissa M. Jordan

**Affiliations:** ^1^Leonard M. Miller School of Medicine, University of Miami, Miami, FL, United States; ^2^Florida Department of Health, Tallahassee, FL, United States

**Keywords:** construction workers, technology, cell phones, construction, vulnerable worker population, temporary workers

## Abstract

**Background:** With high rates of temporary workers and a transient worker population, the U.S. construction workforce presents a challenge for long-term research and outreach activities. Increasing availability of affordable cell phone technologies may provide an opportunity for research follow-up among construction workers once they leave the worksite. Using pilot study survey data we characterize and examine the association of cell phone technology ownership and cellular text/email services among a non-probabilistic sample of payroll and temporary construction workers.

**Methods:** A cross-sectional study design was used to administer a one-time paper-based anonymous survey to construction workers working at construction sites in Florida, USA. The survey featured questions on sociodemographic characteristics, occupational history, cell phone technology ownership, and cellular text/email services capabilities.

**Results:** Among the 223 construction worker survey respondents, 31.4% identified as temporary workers and 68.6% were on payroll and 87.4% owned a cell phone. Construction workers who own a cell phone had greater than a high school education (28.9% vs. 25.0%; *p* = 0.019), made >$30,000/year (27.1% vs. 14.8%; *p* = 0.011), had same cell phone number for >1 year (74.4% vs. 40.7%; *p* = 0.001), and were employed as a payroll worker (71.0% vs. 50.0%; *p* = 0.037). Temporary construction workers compared to their payroll counterparts were significantly less likely to have email services on their cell phone [unadjusted-odds ratio 0.41 (95% CI: 0.17–0.97)].

**Conclusion:** Cell phone ownership and smartphone-enabled technologies such as email/texting capabilities are higher among payroll than temporary construction workers. Further research on frequency of cell phone use and types of email/texting services used by construction workers are needed.

## Introduction

There are well-documented challenges in the recruitment, retention and follow-up of construction workers in both observational and experimental occupational research study designs (1–3). These challenges are more pronounced in the non-union labor force where workers have less social and professional cohesion afforded by the union setting (4). Worker attrition often prevents a full intention to treat analysis and can introduce study bias (5). Strategies to improve construction worker participation in research studies are needed, particularly once they are no longer assigned to a jobsite or employed by the construction firm.

The U.S. Bureau of Labor Statistics estimated that in May 2017, the seasonally adjusted size of the construction workforce was ~7.3 million workers, while temporary construction workers in the same period comprised ~2.9% (215,380 workers) of the total construction workers. The construction industry has grown its use of non-standard employment arrangements and employment through temporary staffing agencies, further challenging approaches to follow-up with construction workers for research studies (6). The rise of low cost communication technologies such as smart phones and pre-paid mobile phone plans in recent years has brought both new opportunities and challenges for occupational health professionals (7–9). Identification of construction worker factors that support consistent and regular engagement in a research project long after they have left the construction site is needed.

In the present study, as a first step to addressing this occupational health and safety research challenge, we conducted a secondary data analysis of self-reported survey data on cell phone ownership and technology access in a non-probabilistic sample of Florida temporary and payroll construction workers collected as part of a larger construction worker research study on injuries. We hypothesized that given the low resources and wages provided to temporary construction workers, they would have less ownership of cell phones, and limited email and text message capabilities as compared to construction workers on a firm's payroll.

## Methods

### Data Source

We conducted secondary analysis of survey data collected in June 2016 from the Falls Reported Among Minority Employees (FRAME) project (2), a cross-sectional study design assessing injuries and near-misses in construction workers. The study included a one-time paper-based anonymous questionnaire administered to construction workers employed across three independent mixed-used residential/commercial building construction sites in South Florida. The 68-item language sensitive (i.e., English and Spanish) survey instrument contained questions organized into sub-sections on worker socio-demographic characteristics; worker training and job tenure; occupational exposures, injuries, near-misses; and on the worker's use of cell phones, email and text messages. Prior to administering the survey instrument, participants were fully explained the study, invited to participated and consented using a verbal consent process. This study was approved by the Institutional Review Board, Biomedical Research Section of the Florida Department of Health (IRB Protocol #:160008U13). The response rate for survey completion was 99.1% (223 completed the survey out of 225 invited workers) of which two construction workers declined to complete the survey due to time constraints at the worksite.

### Survey Measures

Questions on cell phone technology were adapted from standard survey questions available in the U.S. National Health and Nutrition Examination Survey and the Health Information National Trends Survey (10). We included four specific questions: (1) Do you own a cell phone? (response options: Yes / No); (2) How long have you used the same cell phone number? (response options: <1 month, 1 month to 1 year, and more than 1 year); (3) Can you read, send, and receive emails on your cell phone? (response options: Yes/No); and (4) Can you read, send, and receive text messages on your cell phone? (response options: Yes/No).

We defined temporary workers as workers not on the payroll of the general construction contractor or a subcontractor thus, a temporary worker was assigned to the construction jobsite by a temporary staffing agency (11). Payroll workers were defined as those workers who maintain standard work arrangements on the payroll of the contractor or sub-contractor. To categorize workers as either temporary or payroll, the survey instrument included the following question: What type of employment status do you have on your current construction worksite? (response options: temporary worker, worker on payroll with contractor, and don't know/not sure).

### Data Analysis

Frequency and descriptive statistics were calculated for all study variables. Characteristics of temporary workers were compared to payroll workers using the independent sample *t*-test or Mann-Whitney U-test (continuously measured characteristics) or Pearson's Chi square test or Fisher Exact Chi-Square test for two groups (categorical measures). Univariable and multivariable logistic regression models tested the association between employment type (i.e., payroll or temporary construction worker) and the main outcome of worker cell phone email use while controlling for the potential confounders. An alpha level of 0.05 was considered statistically significant. All analyses were conducted using Statistical Package for the Social Sciences (IBM SPSS Statistics for Mac, Version 22.0, Armonk, NY).

## Results

### Sample Demographics

Among the 223 workers completing the paper-based survey at the jobsite, 31.4% self-identified as temporary workers, while 68.6% reported being on the contractor's or sub-contractor's payroll (Table 1). Compared to construction workers who do not own a cell phone, those who do own a cell were significantly more educated with greater than a high school education (28.9% vs. 25.0%; *p* = 0.019), make more than $30,000 per year (27.1% vs. 14.8%; *p* = 0.011), used the same cell phone number for more than a year (74.4 vs. 40.7%; *p* = 0.001), and be employed as a payroll construction worker (71.0% vs. 50.0%; *p* = 0.037).

**Table 1:** Socio-demographic and work-related predictors of cell phone ownership among a sample of construction workers from 3 different jobsites in south Florida (*n* = 223).

**Characteristic**	**Total sample *N* (%)^†^**	**Yes mobile phone ownership *N* (%)^†^**	**No mobile phone ownership *N* (%)^†^**	***p*-value**
**Row Total**	223 (100.0)	195 (87.4)	28 (12.6)	
**Age (in years)**				0.279
Mean Age (SD)	40.4 (10.9)	40.1 (10.6)	42.5 (12.5)	
**Gender**				0.354
Male	215 (97.3)	188 (96.9)	27 (100.0)	
Female	6 (2.7)	6 (3.1)	0 (0.0)	
**Race**				0.217
White	118 (58.7)	99 (56.6)	19 (73.1)	
Black	69 (34.3)	64 (36.6)	5 (19.2)	
Other	14 (7.0)	12 (6.9)	2 (7.7)	
**Ethnicity**				0.317
Non-hispanic	73 (33.3)	66 (34.6)	7 (25.0)	
Hispanic	146 (66.7)	125 (65.4)	21 (75.0)	
**Educational Attainment**				0.019
< High School	50 (22.5)	38 (19.6)	12 (42.9)	
High School/GED	109 (49.1)	100 (51.5)	9 (32.1)	
>High School	63 (28.4)	56 (28.9)	7 (25.0)	
**Individual Income**				0.011
Less than $11,999	67 (30.6)	52 (27.1)	15 (55.6)	
$12,000-$29,999	96 (43.8)	88 (45.8)	8 (29.6)	
More than $30,000	56 (25.6)	52 (27.1)	4 (14.8)	
**Years in Construction Industry**				0.272
Mean Years (SD)	40.4 (9.3)	40.1 (9.2)	42.5 (9.7)	
**Employment Type**				0.037
Temporary Worker	66 (31.4)	54 (29.0)	12 (50.0)	
Payroll Worker	144 (68.6)	132 (71.0)	12 (50.0)	
**Union Membership Status**				0.425
Non-Union	198 (92.5)	172 (92.0)	26 (96.3)	
Union Member	16 (7.5)	15 (8.0)	1 (7.5)	
**OSHA 10 h Training**				0.543
Yes	162 (72.6)	143 (73.3)	19 (67.9)	
No	61 (27.4)	52 (26.7)	9 (32.1)	

† *Differences in sub-total population sample due to item non-response or missing*.

### Univariable and Multivariable Models for Cell Phone Ownership

In the univariable logistic regression analyses (Table 1), temporary construction workers were significantly less likely to report having cell phone ownership compared to payroll construction workers (unadjusted odds ratio, UOR = 0.41; 95% Confidence Interval, CI, [0.17–0.97]). In the multivariable model (Table 2), employment type (i.e., temporary worker status) was not significantly associated with cell phone ownership (Adjusted odds ratio, AOR = 0.25; 95% CI [0.04–1.60]. However, construction workers with high school/GED achievement (vs. less than high school; AOR = 0.34 [0.05–0.99]; individual annual income of $12,000-$29,999 vs. < $11,999; AOR= 0.23 [0.01–0.97]); those who report owning the cell phone number between 1 month and 1 year (vs. <1month) AOR = 0.52 [0.02–0.98], were significantly less likely to own a cell phone.

**Table 2:** Predictors of Cell Phone Ownership among a sample of construction workers from 3 different jobsites in Florida (*n* = 223).

**Predictors**	**Unadjusted model UOR^†^[95% CI]**	**Adjusted model AOR^‡^[95% CI]**
**Employment Type (ref** **=** **Payroll)**		
Temporary Workers	0.41 [0.17–0.97]	0.25 [0.04–1.60]
**Age**		
Age continuous years		1.56 [0.97–3.16]
**Race (ref** **=** **White)**		
Black		0.59 [0.27–2.18]
Other		0.22 [0.02–3.14]
**Ethnicity (ref** **=** **non-Hispanic)**		
Hispanic		1.81 [0.78–2.78]
**Educational Attainment (ref = <HS)**		
High School/GED		0.34 [0.05–0.99]
>High School		1.44 [1.01–2.36]
**Individual Income (ref** **=** **<** **$11,999)**		
$12,000–$29,999		0.23 [0.01–0.97]
>$30,000		1.19 [0.97–2.12]
**Length cell use (ref** **=** **<** **1 month)**		
1 month to 1 year		0.52 [0.02–0.98]
>1 year		1.29 [1.08–1.99]
**Years in construction industry**		
Continuous Years		1.43 [1.11–1.93]
**OSHA 10 h training (ref** **=** **no)**		
Yes		1.48 [0.77–2.36]

† *UOR, Unadjusted odds ratio, the 95% confidence interval, CI*;

‡ *AOR, Adjusted odds ratio, its 95% CI is shown in this table*.

## Discussion

We found that construction workers employed via temporary agencies have less cell phone ownership as compared to workers on the same construction site employed via the construction firm payroll. A 2015 Pew Research Study estimated that 95% of all Americans own a cell phone and 64% own a smartphone; in the 30 to 49 year-old age group alone, the smartphone ownership rate is even higher, at 79% (12, 13). As a vulnerable worker population, temporary construction workers who generally make less wages may be less likely to afford or preferentially pay for a basic cell phone plan and even less so for internet-enabled smartphone technology (14). Nonetheless at the national level, cell phone use has become ubiquitous, particularly among younger adults (18–49 years) (15), suggesting that even temporary workers will experience an increase in the ownership and use of cell phones and smartphones.

Over two-thirds of construction workers in this sample in this study have longer ownership of the same telephone number. Length of ownership of the same number was associated with a higher rate of cell phone email services availability use suggesting that some construction workers perhaps purchase “pay as you go” mobile phone plans and keep the same number longer, at least in a “1 month to 1 year” interval. Access to long-term cell phone use is incredibly helpful to occupational health and safety researchers interested in following up with temporary construction workers once their temporary assignment on a specific worksite has ended. A recent study by Sparer et al., found that close to 60% of largely unionized construction workers remain onsite for at least 1 month (16). Presumably temporary construction workers will spend less time than payroll workers, thus cell phones may provide a mechanism in which to engage and survey temporary workers once they leave the temporary work assignment.

This study is not without limitations. We used a cross-sectional study design to characterize cell phone ownership and smart phone technology availability in a non-probabilistic sample of construction workers. Cell phone ownership may vary throughout the year, and this one-time survey does not capture seasonal variation in cell phone ownership. The study relies on self-report and our research team did not verify if the worker had a cell phone or whether they used email or text messages services, only if they had the service on their phone. Despite these limitations, this pilot study now adds to the extant literature on the ownership of cell phones between temporary and payroll construction workers. This is the first study to characterize smart phone technology ownership among payroll and temporary workers. Leveraging this technology can assist occupational health and safety researchers with surveying and possibly delivering health promotion or safety messaging to this worker group.

Cell phone ownership varies by type of employment arrangement on a construction site. Temporary construction workers own both cell phones and smartphone technologies that could be leveraged for occupational health and safety research. Length of cell phone number ownership was higher for construction workers who owned their cell phone. Given the lower cell phone ownership rates among temporary workers, strategies to increase ownership could support a mechanism to reach and engage this volunteer temporary workforce once they are no longer at the construction jobsite. Further research into seasonal variation in cell phone ownership is needed to understand if cell phones can be used to survey workers more than a year after they have been engaged by the research team at a worksite. Cell phones and smartphones provide promise as a tool to both survey and provide occupational health and safety messaging in hard to reach worker groups but identical research into this methodology is needed.

## Data Availability Statement

The datasets generated for this study are available on request to the corresponding author.

## Ethics Statement

This study has been approved by the Institutional Review Board, Biomedical Research Section of the Florida Department of Health (IRB Protocol #:160008U13).

## Author Contributions

KS, JC, KM, MB, MJ, and AC-M conceived the study, participated in its design, coordination, performed statistical analyses, and co-drafted the manuscript. KM and KS collected field data, entered study data, assisted in data analysis, and interpretation of study results. KS and AC-M performed statistical analysis and helped with the manuscript draft. All authors read, revised, and approved the final manuscript.

### Conflict of Interest

The authors declare that the research was conducted in the absence of any commercial or financial relationships that could be construed as a potential conflict of interest.
